# Trabecular Bone Score Reflects Trabecular Microarchitecture Deterioration and Fragility Fracture in Female Adult Patients Receiving Glucocorticoid Therapy: A Pre-Post Controlled Study

**DOI:** 10.1155/2017/4210217

**Published:** 2017-01-03

**Authors:** Mei-Hua Chuang, Tzyy-Ling Chuang, Malcolm Koo, Yuh-Feng Wang

**Affiliations:** ^1^Department of Pharmacy, Dalin Tzu Chi Hospital, Buddhist Tzu Chi Medical Foundation, Dalin, Chiayi, Taiwan; ^2^Department of Nuclear Medicine, Dalin Tzu Chi Hospital, Buddhist Tzu Chi Medical Foundation, Dalin, Chiayi, Taiwan; ^3^School of Medicine, Tzu Chi University, Hualien City, Taiwan; ^4^Department of Medical Research, Dalin Tzu Chi Hospital, Buddhist Tzu Chi Medical Foundation, Dalin, Chiayi, Taiwan; ^5^Dalla Lana School of Public Health, University of Toronto, Toronto, ON, Canada

## Abstract

A recently developed diagnostic tool, trabecular bone score (TBS), can provide quality of trabecular microarchitecture based on images obtained from dual-energy X-ray absorptiometry (DXA). Since patients receiving glucocorticoid are at a higher risk of developing secondary osteoporosis, assessment of bone microarchitecture may be used to evaluate risk of fragility fractures of osteoporosis. In this pre-post study of female patients, TBS and fracture risk assessment tool (FRAX) adjusted with TBS (T-FRAX) were evaluated along with bone mineral density (BMD) and FRAX. Medical records of patients with (*n* = 30) and without (*n* = 16) glucocorticoid treatment were retrospectively reviewed. All patients had undergone DXA twice within a 12- to 24-month interval. Analysis of covariance was conducted to compare the outcomes between the two groups of patients, adjusting for age and baseline values. Results showed that a significant lower adjusted mean of TBS (*p* = 0.035) and a significant higher adjusted mean of T-FRAX for major osteoporotic fracture (*p* = 0.006) were observed in the glucocorticoid group. Conversely, no significant differences were observed in the adjusted means for BMD and FRAX. These findings suggested that TBS and T-FRAX could be used as an adjunct in the evaluation of risk of fragility fractures in patients receiving glucocorticoid therapy.

## 1. Introduction

Osteoporosis is a well-defined systemic disorder characterized by low bone mass accompanied by a microarchitecture weakening of the bone tissue, with a subsequent increase in bone breakability [1–5]. The diminished bone density associated with this disease is a major risk factor for fractures, especially fractures of the hip, spine, and wrist. Osteoporosis is primarily a consequence of physiological bone loss, but it can be secondary to certain medical treatment (e.g., glucocorticoid (GC), anticonvulsants, cytotoxic drugs, excessive thyroxine, heparin, aluminum-contained antacids, lithium, and tamoxifen) or diseases, such as rheumatoid arthritis, diabetes, chronic kidneys, and primary hyperparathyroidism [6–8].

Long-term use of GC is frequent among patients with various systematic diseases, such as rheumatoid arthritis, systemic lupus erythematosus, inflammatory bowel diseases, and chronic obstructive lung diseases [7, 9]. However, GC use can affect mineral metabolism in bone cells, damage coupling activities of bone formation and resorption, promote osteoblasts apoptosis, inhibit osteoblasts propagation, and synthesize type I collagen and osteocalcin [10–12]. In addition, GC can reduce intestinal absorption of calcium, while increasing calcium excretion from the kidneys, causing an increase in parathyroid hormone secretion. All of these together can lead to significant damage to the bone tissue of vertebral and nonvertebral bones [13, 14], leading to the development of GC-induced osteoporosis (GIO). Previous studies have shown that fractures occur in 30%–50% of patients receiving long-term GC therapy [15]. Moreover, patients receiving GC therapy have an increased risk of fracture at a higher level of bone mineral density (BMD) value compared to patients who were not receiving GC therapy [16, 17].

The BMD value, acquired with a dual-energy X-ray absorptiometry (DXA) scanner, is an estimation of the quantity of the bone. A low BMD value is inversely proportional to an increase in fracture risk [5, 18]. Only quantitative information can be produced from the two-dimensional DXA images (i.e., areal BMD) and no qualitative three-dimensional information relating to bone structure can be obtained from BMD alone. However, microarchitectural and qualitative properties must also be considered when assessing the ability of bone to resist fracture. Therefore, BMD values may not be able to adequately reflect the increased fracture risk related to alterations in bone microstructure among patients receiving long-term GC therapy [19, 20]. Similarly, while fracture risk assessment tool (FRAX) can be used to predict the 10-year probability of a major osteoporotic fracture, such as spine, hip, forearm, or humorous fractures [21], many fragility fractures occur in osteopenic individuals (*T*-score between −1.0 and −2.5) rather than just in those with osteoporosis (*T*-score < −2.5) [22]. Consequently, factors such as bone geometry and bone microarchitecture are important in determining risk of fractures in addition to BMD.

A recently developed diagnostic tool, trabecular bone score (TBS), can provide quantified information on trabecular microarchitecture [23–28] using gray-level texture measurements of lumbar spine DXA images. Since TBS can be extracted from DXA images of the lumbar spine, it can readily be retrospectively applied to existing DXA images obtained from the majority of standard DXA devices. Therefore, TBS can provide additional skeletal information that is not available in standard BMD measurement. An elevated TBS value indicates a stronger skeletal texture, which is a reflection of better microarchitecture. Existing literature indicated that the use of TBS is valuable in predicting the associations between fragility fractures in healthy women [29, 30] and in patients with various clinical disorders, such as rheumatoid arthritis, diabetes, chronic kidneys, and primary hyperparathyroidism [31–33]. Moreover, an improvement in fracture prediction capacity has been documented when TBS was used to adjust FRAX (T-FRAX) [34]. To this end, the aim of this study was to evaluate the performance of TBS and T-FRAX in comparison to BMD and FRAX for predicting fracture risk in adult female patients receiving GC treatment.

## 2. Materials and Methods

### 2.1. Study Population

Medical records, during the period from 2011 to 2014, from a regional hospital in southern Taiwan were retrospectively reviewed. Adult female patients (40 to 89 years old) who underwent BMD measurements by DXA were identified. Only patients who had received two DXA scans of the lumbar spine and bilateral hip areas within an interval of 12 to 24 months were included in the study. For the exclusion criteria, patients who had undergone a surgical procedure to the spinal vertebrae or hips, such as internal fixation or total hip replacement, and diseases related to secondary osteoporosis, including type-2 diabetes, hyperparathyroidism, and hypercortisolism, were excluded from the study. In addition, patients who had received the following concomitant medications were excluded: tamoxifen > 5 years prior to baseline; lithium > 2 years prior to baseline; carbamazepine, phenobarbital, or proton pump inhibitors > 1 year prior to baseline, phenytoin, or heparin > 3 months prior to baseline; and cyclophosphamide or high-dose (≥500 mg/m^2^) methotrexate > 1 month prior to baseline.

A total of 46 women were included in this study and they were divided into two groups based on their GC use. The GC group (*n* = 30) comprised of patients receiving glucocorticoid therapy, while the non-GC group (*n* = 16) was comprised of patients without receiving GC therapy. The latter group consisted of patients who had undergone routine health examinations at the study hospital.

### 2.2. DXA, BMD, and TBS Assessments

Areal BMD of the lumbar spine (vertebrae L1–L4) was measured with DXA (Discovery Wi, Hologic Inc., Boston, MA, USA). TBS values of the same lumbar vertebrae were determined based on DXA images using dedicated analysis software (TBS iNsight, version 2.1.2.0, Medimaps, Mérignac, France).

### 2.3. FRAX Measurements and Fracture Risk Assessments

The FRAX [35], developed by the World Health Organization Collaborating Centre for Metabolic Bone Diseases, provides estimates for a 10-year probability of major and hip osteoporotic fracture [36]. A higher risk value indicates the necessity for treatment, whereas a low risk value suggests that only a follow-up is required. Fracture risk was assessed for all patients using the online FRAX tool provided by the Centre for Metabolic Bone Diseases at Sheffield University (http://www.shef.ac.uk/FRAX/tool.jsp). Furthermore, T-FRAX was also obtained using an online tool (http://www.shef.ac.uk/TBS/CalculationTool.aspx).

### 2.4. Statistical Analysis

All results are expressed as mean ± standard deviation except indicated otherwise. Comparisons between the basic characteristics of the patients with and without GC therapy at baseline were analyzed by *t*-test and Fisher's exact test for continuous variables and categorical variables, respectively. Comparisons of BMD, TBS, FRAX, and T-FRAX between baseline and follow-up (within group) were based on the percentage changes of baseline value (Δ = 100 × [follow-up value − baseline value]/baseline value). One-sample *t*-test was used to evaluate whether Δ was significantly different from 0. In addition, analysis of covariance (ANCOVA) was used to compare BMD, TBS, FRAX, and T-FRAX between the GC group and the non-GC group, adjusting for age and baseline values. Results of ANCOVA are presented as least-squares adjusted means with 95% confidence intervals. All statistical analyses were conducted using SPSS software, version 24.0 (IBM Corp., Armonk, NY, USA). A *p* value < 0.05 was considered statistically significant.

## 3. Results

### 3.1. Basic Characteristics of the Study Patients

The baseline characteristics of the patients are shown in Table 1. Age was significantly younger in the GC group (*p* = 0.047). The proportion of patients with fractures was significantly lower in the GC group (*p* < 0.001). There were no significant differences in height, weight, body mass index, follow-up period, and the proportion with rheumatoid disease between the two groups.

### 3.2. Changes in Bone Mineral Density between Baseline and Follow-Up

**Figure 1:**
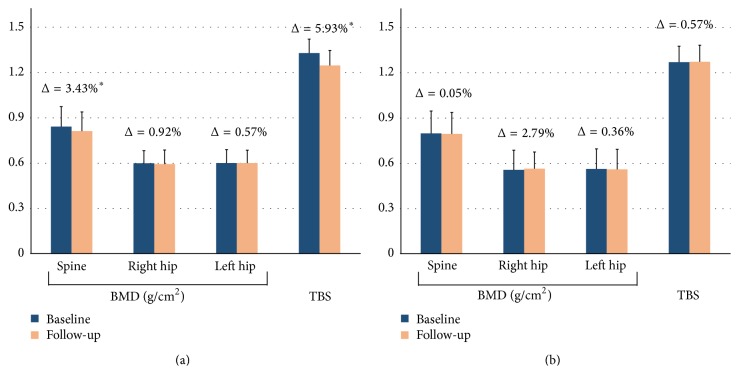
Bone mineral density (BMD) and trabecular bone score (TBS) values of the detected areas at baseline (blue) and follow-up period (orange) in the GC (a) and non-GC (b) groups. Values shown above the data bars are Δ, calculated as 100 × (follow-up value − baseline value)/baseline value. ^*∗*^*p* < 0.05.

Table 2 shows the percentage changes of BMD over time analyzed separately for the GC and non-GC group. In the GC group, there was a significant decline in the average lumbar spine BMD from baseline to follow-up (*p* = 0.004), with a Δ of −3.43. The change in TBS (Δ = −5.93, *p* < 0.001) over time was also significant with a larger magnitude of Δ than that of BMD. No significant differences in the percentage change over time in the right and left hip BMD were observed. In the non-GC group, neither BMD nor TBS showed significant differences in their percentage changes over time (Figure 1).

### 3.3. Assessment of Fracture Risk

**Figure 2:**
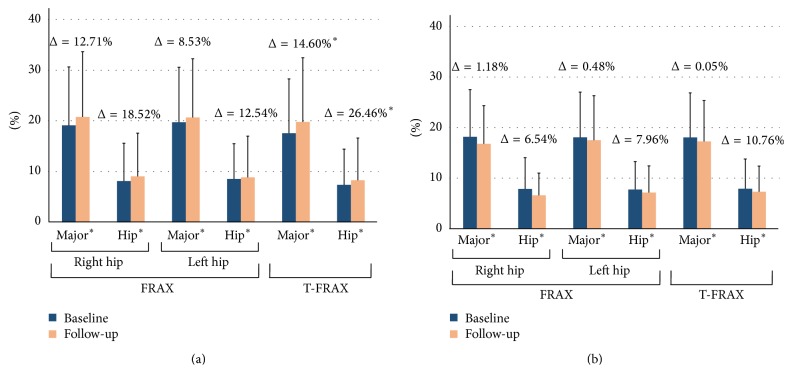
Fracture risk at baseline (blue) and follow-up period (orange) in the GC (a) and non-GC (b) groups. Fracture risks for major osteoporotic (labeled as “major^*∗*^”) and hip fractures (labeled as “hip^*∗*^”) were measured using the FRAX tool and with FRAX adjusted for TBS (T-FRAX). Values shown above the data bars are Δ, calculated as 100 × (follow-up value − baseline value)/baseline value. ^*∗*^*p* < 0.05.

For the assessment of fracture risk in the GC group, T-FRAX exhibited a significant increase in both the risk for major osteoporotic fracture (Δ = 14.60, *p* < 0.001) and hip fracture (Δ = 26.46, *p* = 0.001). On the other hand, no significant differences were observed in the percentage change of FRAX over time. For the assessment of fracture risk in the non-GC group, neither the percentage change in FRAX nor T-FRAX was significantly different between baseline and follow-up (Figure 2).

### 3.4. Comparison of Age- and Baseline-Adjusted Means at Follow-Up between the GC and Non-GC Group

Results from ANCOVA (Table 3) indicated that the age and baseline value adjusted mean of TBS was significantly lower in the GC group (*p* = 0.035). In addition, the age and baseline value adjusted mean T-FRAX for major osteoporotic fracture was significantly higher in the GC group (*p* = 0.006). The age and baseline value adjusted mean T-FRAX for hip fracture was marginally higher in the GC group (*p* = 0.056). Conversely, no significant differences were observed in the age and baseline value adjusted mean for BMD and FRAX between the two groups.

## 4. Discussion

In this pre-post controlled study comparing patients with and without GC therapy, our results revealed that the lumbar vertebrae showed a statistically significant decline over time in the GC group (Table 2). In contrast, both BMD for the hip areas and FRAX did not show significant percentage changes between baseline and follow-up. This finding is consistent with the notion that the use of oral GC is generally associated with an impairment of the microarchitectural texture at the central skeleton but not BMD at other sites [37]. On the other hand, we found a significant decrease in the percentage change in TBS for lumbar spine and an increased risk (T-FRAX) for major fracture and hip fracture. This observation is also consistent with findings from previous studies that TBS could differentiate between GC-treated and non-GC-treated female patients [20]. In addition, in the GC group, the magnitude of percentage change in TBS was approximately twice as large as that in the BMD value (−5.93%* versus* −3.43%). This pattern was not apparent in the non-GC group, neither in TBS nor in T-FRAX, in comparison to that of BMD or FRAX. This suggests that TBS and T-FRAX appeared to provide an augmented risk evaluation for fragility fractures in long-term GC-treated female patients.

Another important finding in this study is that significant differences at follow-up were observed in TBS and T-FRAX (major osteoporotic fracture) in the GC group compared with the non-GC group. On the other hand, no significant differences between the two groups were observed in BMD or FRAX at the follow-up (Table 3). The effects of different age and baseline values were adjusted in these comparisons using ANCOVA. This finding further demonstrated the potential value of TBS and T-FRAX over BMD alone for assessing the risk of fragility fractures.

Our study has several limitations that deserve mention. First, TBS is not a direct physical measurement of the bone microarchitecture. Factors affecting the digital radiography image quality can influence the accuracy of TBS measurements. Image acquisition noise, such as X-ray quantum noise, detector defects, quantization noise, or scatter radiation, can lead to DXA image resolution degradation and therefore affecting TBS estimation. Second, our TBS results may not be comparable with other studies using different DXA machines. Third, our data were based on records from a single hospital, which may limit the generalizability of our results. Fourth, our study design is observational and therefore the possibility of confounding by unmeasured variables cannot be completely ruled out.

Despite the aforementioned limitations, there are also important strengths in this study. To our knowledge, this is the first study that used a longitudinal design to assess the changes in BMD and TBS over time in female patients receiving GC. Our findings demonstrated that TBS and T-FRAX were able to detect changes over time in bone quality that was not apparent from BMD measurement alone. An enlarged effect of percentage changes over time in TBS over BMD in predicting the bone mineral differences was also observed, which provide support for the use of TBS as an adjunct measurement for assessing the risk of fragility fractures.

## 5. Conclusion

Bone quality, in addition to quantity, plays an important role in the risk of fragility fractures. Patients receiving GC therapy usually experience significantly diminished BMD values and increased fracture risk, as measured by FRAX. Findings from our study revealed that TBS and T-FRAX showed an amplified predictive ability for osteoporotic fracture risk assessment in patients receiving GC therapy. Therefore, from a clinician's point of view, TBS and T-FRAX, which can readily be obtained from DXA images, should be considered as an adjunct tool for the risk evaluation of fragility fractures in patients receiving GC therapy.

## Figures and Tables

**Table 1:** Basic characteristics of female patients with and without glucocorticoid therapy (*N* = 46).

Variable	GC group (*n* = 30)	Non-GC group (*n* = 16)	*p*
Age (years)	62.9 ± 13.0	71.1 ± 12.9	0.047
Height (cm)	152.5 ± 5.5	151.4 ± 7.6	0.555
Weight (kg)	55.6 ± 8.3	51.6 ± 9.4	0.146
Body mass index (kg/m^2^)	23.9 ± 3.4	22.4 ± 2.7	0.129
Follow-up period (months)	14.6 ± 6.5	18.6 ± 9.2	0.141
Rheumatoid disease, *n* (%)	4 (13.3)	0 (0)	0.282
Fractures, *n* (%)	5 (16.7)	13 (81.3)	<0.001

*p* values were obtained from Fisher's exact test for categorical variables and *t*-test for continuous variables. Data are expressed as mean ± standard deviation unless otherwise indicated.

**Table 2:** Comparison of bone mineral density, trabecular bone score, and fracture risk assessment tool score between baseline and follow-up for patients with and without glucocorticoid therapy (*N* = 46).

Variable	Glucocorticoid group (*n* = 30)				Nonglucocorticoid group (*n* = 16)			
	Baseline	Follow-up	Δ (%)	*p* for Δ	Baseline	Follow-up	Δ (%)	*p* for Δ
BMD (g/cm^2^)								
Lumbar spine	0.8423 ± 0.1324	0.8120 ± 0.1272	−3.43 ± 6.03	0.004	0.7986 ± 0.1486	0.7951 ± 0.1427	−0.05 ± 7.02	0.977
Right hip	0.5996 ± 0.0831	0.5940 ± 0.0932	−0.92 ± 7.76	0.519	0.5575 ± 0.1303	0.5654 ± 0.1106	2.79 ± 8.6	0.214
Left hip	0.6004 ± 0.0900	0.6011 ± 0.0851	0.57 ± 7.76	0.691	0.5632 ± 0.1327	0.5608 ± 0.1330	−0.36 ± 9.12	0.876
TBS	1.3285 ± 0.0928	1.2467 ± 0.0996	−5.93 ± 7.54	<0.001	1.2703 ± 0.1057	1.2723 ± 0.1101	0.57 ± 10.04	0.822
FRAX (%)								
Right, major fracture	19.11 ± 11.51	20.77 ± 12.87	12.71 ± 41.38	0.103	18.17 ± 9.34	16.78 ± 7.55	−1.18 ± 19.86	0.815
Right, hip fracture	8.12 ± 7.46	9.05 ± 8.53	18.52 ± 55.89	0.080	7.86 ± 6.17	6.58 ± 4.42	6.54 ± 69.34	0.711
Left, major fracture	19.73 ± 10.83	20.67 ± 11.56	8.53 ± 29.48	0.124	18.08 ± 8.94	17.51 ± 8.79	0.48 ± 17.86	0.916
Left, hip fracture	8.54 ± 6.94	8.86 ± 8.13	12.54 ± 43.37	0.124	7.75 ± 5.52	7.14 ± 5.28	7.96 ± 44.81	0.488
T-FRAX (%)								
Major fracture	17.56 ± 10.70	19.79 ± 12.65	14.60 ± 16.48	<0.001	18.04 ± 8.82	17.25 ± 8.08	−0.05 ± 18.40	0.991
Hip fracture	7.37 ± 7.06	8.29 ± 8.31	26.46 ± 37.68	0.001	7.89 ± 5.90	7.31 ± 5.07	10.76 ± 43.87	0.342

BMD: bone mineral density; FRAX: fracture risk assessment tool; TBS: trabecular bone score; T-FRAX: TBS-adjusted FRAX;

Δ: 100 × (follow-up value − baseline value)/baseline value.

*p* values were obtained from one-sample *t*-test of Δ.

Data are expressed as mean ± standard deviation.

**Table 3:** Comparison of age-adjusted bone mineral density, trabecular bone score, and fracture risk assessment tool score of female patients with and without glucocorticoid therapy at follow-up (*N* = 46).

Variable	Age- and baseline-adjusted mean (95% confidence interval)		*p*
	GC group (*n* = 30)	Non-GC group (*n* = 16)	
BMD (g/cm^2^)			
Lumbar spine	0.797 (0.778–0.817)	0.822 (0.796–0.849)	0.138
Right hip	0.580 (0.564–0.596)	0.591 (0.569–0.613)	0.445
Left hip	0.588 (0.574–0.603)	0.585 (0.564–0.605)	0.785
TBS	1.233 (1.199–1.267)	1.298 (1.250–1.346)	0.035
FRAX (%)			
Right, major fracture	20.23 (17.93–22.52)	17.79 (14.50–21.09)	0.257
Right, hip fracture	8.90 (7.43–10.38)	6.86 (4.78–8.93)	0.127
Left, major fracture	20.41 (18.27–22.54)	18.00 (14.93–21.07)	0.231
Left, hip fracture	8.60 (7.27–9.92)	7.63 (5.76–9.50)	0.419
T-FRAX (%)			
Major fracture	20.01 (18.78–21.24)	16.83 (15.10–18.57)	0.006
Hip fracture	8.52 (7.58–9.45)	6.89 (5.58–8.20)	0.056

BMD: bone mineral density; FRAX: fracture risk assessment tool; TBS: trabecular bone score; T-FRAX: TBS-adjusted FRAX.

*p * values comparing the two groups were obtained from analysis of covariance, adjusted for age and baseline values.
